# Child rearing knowledge and practice scales for women with epilepsy

**DOI:** 10.4103/0972-2327.70877

**Published:** 2010

**Authors:** P. P. Saramma, Sanjeev V. Thomas

**Affiliations:** Kerala Registry of Epilepsy and Pregnancy, Department of Neurology, Sree Chitra Tirunal Institute for Medical Sciences and Technology, Trivandrum - 695011, Kerala State, India

**Keywords:** Child rearing knowledge, child rearing practice scale, reliability and validity

## Abstract

**Background:**

Comprehensive instruments to evaluate the child rearing knowledge and practice are not readily available for clinical research.

**Materials and Methods:**

We have designed in two phases a new instrument to evaluate the child rearing knowledge and practice under the four major domains of child rearing. Twenty-five subject experts from the field of Paediatrics, Obstetrics, Neurology and Nursing elicited the content validity of the instrument. The test retest reliability was evaluated by 25 young mothers who completed the CRKS at an interval of two weeks.

**Results:**

The Content Validity Ratio (CVR) of individual items ranged between 0.6 to 1. The reliability was tested for the 20 individual items of the CRKS using Kappa coefficient. The measurement of agreement Kappa ranged from 0.51 to 1. The total knowledge scores and sub scores data were analysed for correlation using Pearson’s correlation coefficient. A significant Pearson’s correlation indicated that the total scores were consistent over time (r = 0.89). The sub scores on feeding (6 items), Growth and development (4 items), protection (7 items), and infant stimulation (3 items) were found to have reliability of 0.91, 0.76, 0.84, and 0.89 respectively using Pearson’s correlation.

**Conclusion:**

The instrument is found to be valid and reliable and can be used to measure child rearing knowledge and practice in early infancy.

## Introduction

Child rearing (CR) refers to bringing-up of children by parents or parent substitutes. It consists of practices that are grounded in cultural patterns and beliefs. It is probably the most challenging responsibility for a mother during her child’s infancy. Successful CR is essential for the child’s overall development and realization of self-esteem.[1] As the primary care giver for infant, mother is responsible for attending to all the needs of the infant. In India, other elder members of the family also contribute to childcare. The important components of CR are maternal activities that promote the children’s physical, intellectual, and psychosocial development so that they may grow up to express their full potentials.

The major domains of CR during infancy are feeding, meeting the needs of cleaning, and protection including prevention of accidents and injuries, providing appropriate infant stimulation, and monitoring growth and development. Child rearing practices (CRP) are influenced by child rearing knowledge (CRK). Women with chronic diseases such as epilepsy may have limitations in CR but appropriate interventions can improve baby outcome.[2–4] Women with epilepsy have poor CRK. Women with frequent seizures may find it difficult to practice CR safely and efficiently and should solicit help and support from family members. By systematically evaluating the CR knowledge and practices, it is possible to identify areas of inadequacy and institute remedial programs, and thereby ensure proper growth and development of the babies. There have been few studies that had comprehensively evaluated CR. Until recently, the entire attention of the medical fraternity had been focused on to breastfeeding. There are several instruments that evaluate the breastfeeding habits of mothers. Nevertheless, there are no scales that examine comprehensively all domains of child rearing. Broad based scales are necessary for evaluating efficacy of any CR intervention programs.

An earlier scale that was used in field trials in Ghana had three indices: a child-feeding index; a preventive health seeking index; and a hygiene index.,[5] another scale consisted of only agespecific child-feeding index.[6] These indices did not evaluate the domains of infant stimulation or protection. The investigators experienced the urgent need for a comprehensive scale to evaluate child rearing knowledge and practices applicable to infancy (up to 1 year of age) while attempting to evaluate potential intervention programs for women with epilepsy. The objective of this study was to test the validity and reliability of a newly developed comprehensive scale to evaluate child rearing knowledge and practice in mothers of infants.

## Materials and Methods

### Setting

This scale was prepared in the Kerala Registry of Epilepsy and Pregnancy in the Sree Chitra Tirunal Institute for Medical Sciences and Technology, in order to evaluate the child rearing knowledge and practice of women with epilepsy. We prepared a draft scale after carrying out an extensive literature survey, consultations with the national guidelines on infant feeding, discussions with experts and mothers on prevailing CR norms. The questions were assembled from the four domains of child rearing. The normative data for infant development was based on Simplified Developmental Information Chart.[7] These scales were assessed in a pilot study in our institute.[8] The final version that we prepared had two parts: Child Rearing Knowledge Scale (CRKS) and Child Rearing Practice Scale (CRPS).

### The child rearing knowledge scale

The CRKS is designed for administration to women during pregnancy or in post-partum period up to 4 months. This selfreporting type of scale has 20 multiple choice questions on infant care divided into four subscales that covered the four domains of CR viz. feeding (questions 1-6), growth and development (questions 7-10), cleaning and protection (questions 11-17), and infant stimulation (questions 18-20). Each question had five choices: one right choice, three wrong choices, and a ‘don’t know’ choice to avoid the chance of guessing. The correct choice carried two points and other choices were scored as ‘0’. The range of score for an individual was between 0 and 40. Higher score meant better CRK. The scale is prepared in the vernacular language (Malayalam) and is designed for self-administration in 20 min time. An English translation of the scale is provided in Appendix 1. Mothers were expected to answer the questions all by themselves. The relatives and bystanders were advised not to assist the subject during the administration of the test.

### The child rearing practice scale

This section of the scale is designed for administration by the investigator in a face-to-face interview (Appendix 2). It consisted of 25 items divided into 4 subscales, that covered the four major child rearing domains related to early infancy and related practices viz. feeding (item 1-7), growth and development (items 8-9), cleaning and protection (items 10-21), and infant stimulation (items 22-25). The domain-cleaning and protection was further sub-classified into hygiene of the baby (items 10-14), infection prevention (items 15-17), and prevention of accidents and injuries of the baby (items 18-21). The items were scored based on the reported behaviors of mothers on these four domains. Out of the 25 maternal behaviors in the CRPS, 3 were rated on a four-point scale and the remaining 22 were dichotomous Yes/No questions. The total CRP score was calculated as the sum total of the four subscale scores and ranged from 0 to 34. Higher scores indicated better child rearing practices. The CRP Scale could be administered in 20 min time.

### Testing the scales for content validity and reliability

#### Validity

In the first phase, the draft CRKS was circulated among subject experts and their opinion was taken. The panel of subject experts were from nursing (four postgraduate nursing faculty, two clinical bedside nurses), pediatrics (two teaching faculty with special interest in child development and nutrition), and obstetrics (two postgraduate teaching obstetricians who regularly attend to women with special problems such as epilepsy). Their recommendations and suggestions were incorporated in the instrument.

In the second phase, CVR was determined by distributing the instruments to 30 subject experts including specialists from six institutions. Twenty-seven of them returned the instruments after validation along with their comments, two of whom were excluded because of incomplete data. There were 19 female experts and 6 male experts, which included neurologists (*n*=4), pediatricians including one pediatric neurologist (*n*=4), gynecologists (*n*=2), and 5 each from the field of pediatric nursing, obstetric nursing, and community nursing (*n*=15). Their professional experience ranged from 2 to 27 years with a mean of 16.36 ± 8.5 years. Each expert was asked to evaluate on a four-point ordinal scale whether or not the individual items in the CRKS (20 items) and the CRPS (25 items) were indeed relevant in measuring the four domains of CR. Items which were rated as “1” corresponded to “least relevant” and a rating of “4” corresponded to “highly relevant.” Items rated as 3 or 4 were considered relevant and were included in the final version of the scales after calculating the CVR. The method for determining CVR as developed by Lawshe[9] is described by the following formula:

$$\mathrm{CVR}\;=\;\frac{\mathrm{ne}\;-\;N/2}{N/2}$$

where CVR is the Content Validity Ratio, ne is the number of panellists indicating useful/relevant about a specific item, and *N* is the total number of panellists. The minimum value of the CVR to be significant with 25 panelists is 0.37 per identified item.

#### Reliability

Reliability of the scales was evaluated by the test-retest method by administering the test at 2 weeks interval to 25 employed women [Table 1]. Their ages ranged between 21 and 55 with a mean (SD) of 29.9 (9.9) and were working as nurses (19), doctors (2), or clerks (4). The subjects were told the purpose of the study and requested not to review or discuss related information during the 2 weeks interval period and assured that the questions and the correct answers would be discussed with them after the retest. The same test was repeated after 2 weeks. None of them reviewed related materials during this period. All 25 women completed the second test. The results of the two tests were analyzed for measurement of agreement using Kappa coefficient. The total knowledge scores and sub-scores data were analyzed for correlation using Pearson’s correlation coefficient.

**Table 1:** Demographic characteristics of subjects who participated in the reliability test *n*=25

Characteristics	*n*	%
Age (years)		
21-30	17	68
31-40	2	8
41-50	4	16
>50	2	8
Marital status		
Married	15	60
Unmarried	10	40
Religion		
Hindu	13	52
Christian	11	44
Muslim	1	4
Education		
12 years of schooling	2	8
15 years of schooling	2	8
Medical	2	8
Nursing	19	76
Presence of infants/toddlers at home		
Yes	10	40
No	15	60
Total	25	100

## Results

### Content validity ratio

The CVR was calculated as a means of quantifying the degree of consensus among the panel of 25 experts, who evaluated the scales for content validity. The CVR according to the expert panellists ranged from 0.6 and 1.0 for the CRKS [Table 2] as well as the CRPS [Table 3]. The experts had judged that the instrument had good face validity and that all the items were relevant. We prepared the final scales by making certain minor modifications in the wordings and language according to the suggestions of the experts.

**Table 2:** Child rearing knowledge scale: Content validity ratio according to expert panellists and Kappa coefficient for the reliability test

Items	Judged as relevant by experts (*n*=25)	CVR	Kappa value
The first food to be given to a newborn baby is ___	25	1.0	0.779*
When should breastfeeding be initiated after a normal delivery?	25	1.0	0.781*
How long should breastfeeding be continued?	24	0.92	0.759*
Which of the following breast parts should be inside the baby’s mouth in correct sucking position?	24	0.92	0.915*
What is the correct time for beginning complementary feeding?	23	0.84	0.679*
A 1-year-old infant can be given ___	23	0.84	0.651*
When does a baby’s weight become approximately double the birth weight?	24	0.92	0.675*
Normally, when does a baby attain head control?	25	1.0	0.911*
Normally when does a baby start sitting without support?	25	1.0	0.513*
Normally when does a baby start saying syllables like ‘da’, ‘pa’, etc?	24	0.92	0.781*
How can you safeguard a baby against infections?	24	0.92	0.359
How can you ensure safety of the baby while bathing him or her?	22	0.76	0.339
What is the ideal way to clean the baby’s clothes?	21	0.68	1.000*
Which of the following accidents can occur to an infant?	25	1.0	0.752*
How can you prevent possible suffocation in infants?	25	1.0	1.000*
Which one of the following immunizations needs not be repeated during infancy?	20	0.6	1.000*
If the baby gets diarrhea how will you modify his feeds?	24	0.92	0.896*
A baby explores the world through	24	0.92	0.750*
Which one of the following is most helpful for the mental development of the baby?	23	0.84	**
Language stimulation can be provided to the baby by	23	0.84	**

* Correlation is significant at the 0.00 level;

** Kappa could not be calculated because there was 100% agreement.

**Table 3:** Child rearing practice scale: Content validity ratio according to expert panellists (*n*=25)

Items	No of experts who had judged the question as relevant	CVR
Did you give the first milk (colostrum) to your baby?	25	1
Were you able to timely initiate breastfeeding within half an hour if normal delivery/ within 4 hours if Caesarian?	25	1
Are you currently breast feeding your baby?	23	0.84
What type of milk feeds are you giving to your baby? Exclusive breast feeds=4, BF+Bottle feed =3, Bottle feed only =2, Bottle feed +animal milk =1	25	1
Have you started complimentary feeding (–ve item)?	24	0.92
How often are you able to meet the feeding needs of your baby? Always =4; Often =3; Occasionally =2; Rarely=1	20	0.6
Are you able to meet the needs of your baby during night-time?	22	0.76
Do you check the weight of your baby?	25	1
Do you check for normal development of your baby (social smile, head holding etc)?	25	1
Do you keep the baby clean?	21	0.68
How often do you change the diaper as soon as it is soiled? Always =4; Often =3; Occasionally =2; Rarely=1	23	0.84
Do you bathe the baby by yourself?	24	0.92
Do you groom and dress the baby by yourself?	24	0.92
Do you wash the baby’s dresses with soap and water and dry in sunlight?	24	0.92
Do you wash your hands before caring the baby?	24	0.92
Do you restrict persons with infection from handling your baby?	25	1
Did you give immunizations to your baby on scheduled dates?	24	0.92
Are you in the habit of covering your baby from cold climate?	22	0.76
Is your baby sleeping with you in the same bed/same room during night?	24	0.92
Do you keep your baby on a mat on the floor?	21	0.68
Have your baby met with any accidents (e.g. falling from the cot/cradle/hand, injury from pointed objects)? (–ve item)	24	0.92
Are you able to console your baby when he/she cries?	23	0.84
Do you talk to your baby/introduce family members to him?	25	1
Do you play with your baby using colored/sound making toys?	25	1
Do you sing songs (Lullaby) for your baby to make him sleep?	25	1

### Reliability

The Kappa score for the test–retest reliability test for the CRKS is given in Table 2. The mean knowledge score of healthy women for first test was 28.4 ± 5.5 with a range of 18 to 38 and for the retest it was 30 ± 2.9 with a range of 20 – 40. All the individual items except item 11 and 12 were found to have adequate agreement between the two test scores (the measurement of agreement Kappa ranged from 0.51 to 1). Accordingly these two questions and choices were modified. Kappa coefficient could not be calculated for item number 19 and 20 since there was 100% agreement [Table 2].

The total score had significant consistency over time (r = 0.891) according to Pearson’s correlation. The sub-scores on feeding, growth and development, cleaning and protection, and infant stimulation were found to have reliability of 0.906, 0.758, 0.836, and 0.89, respectively, using Pearson’s correlation coefficient [Table 4].

**Table 4:** Pearson’s correlation coefficient for the sub-domains-CRKS

Domains of child rearing	r
Feeding	0.906*
Growth and development	0.758*
Cleaning and Protection	0.836*
Infant stimulation	0.890*
Total child rearing knowledge score	0.891*

* Correlation is significant at the 0.01 level (two-tailed)

## Discussion

It is important to have reliable instruments to evaluate CRK and CRP. This study had demonstrated that both the instruments (CRKS and CRPS) had strong validity and reliability for measuring child rearing knowledge and practice. The CVR for individual items in both the CRKS and the CRPS ranged from 0.6 to 1, which indicates that each item measures the child rearing knowledge and child rearing practices as intended (Appendices 1 and 2). The criterion-related validity was not estimated due to the non-availability of similar scales.

The instruments included items supported by current evidence-based information, emphasizing WHO guidelines, and Breastfeeding promotion network of India guidelines.[10,11]

The WHO and UNICEF recommend that the infants should be given only breast milk for the first 6 months of their life and then breastfeeding should be continued to 2 years or more along with complementary feeding to achieve optimal growth, development, and quality survival.[12] When the mother is the primary caretaker, mother-infant bonding is established[7] and it is reduced when the care of the baby is undertaken by a third person.

Maternal schooling was the most consistent constraint to all three categories of childcare practices namely child feeding, health seeking, and hygiene practices in Accra.[5] The first two indices were based on data from maternal recall (with children under 3 years of age). The traditional child-feeding practices included breastfeeding, use of prelacteal feedings, and timing of introduction of complementary liquids and foods in the child’s diet. The preventive health seeking behaviors included attendance at growth monitoring and whether the child had been immunized. This index seemed to be a very useful tool for examining associations between childcare and nutritional status.[13] The hygiene index was based on spotcheck observations of proxies for hygiene behaviors. A positive relationship between the child rearing knowledge and child rearing practice was observed in the pilot study.[7]

Ruel and Menon created a child-feeding index including the dimensions of breastfeeding practices, dietary diversity, food frequency, and meal frequency based on DHS data sets from five Latin American countries[6] and the results showed a significant association between the child-feeding index and child-nutritional status. Nevertheless, this composite feeding index was not associated with physical growth among the rural African children.[14] The NFHS-2 data set does provide child feeding information, but not in sufficient detail to prepare such an indicator.[15] A comparison of the available child feeding scales is given in Table 5.

**Table 5:** Comparison of existing scales on child rearing

Scale	Domains evaluated	Remarks
Composite child care index, Accra (Ruel *et al*. 1999)[16]	Traditional child feeding care giver-child interaction preventive health seeking behavior	Comprehensive, but lacks infant stimulation
Child feeding index Latin America Ruel and Menon 2002[6]	Breast feeding Dietary diversity Food/meal frequency	Only feeding aspects
Infant/child feeding index Ethiopia Arimond and Ruel 2002[17]	Breast feeding score Bottle use score Dietary diversity Feeding frequency	Only feeding aspects
Child rearing knowledge and practice scale (present scale in this article)	Feeding Cleaning and protection Infant stimulation Growth and development	Comprehensive Child rearing Scale during infancy

The instruments CRKS and CRPS can be used to assess the child rearing knowledge and practices of normal women and women with special problems such as epilepsy. All items in the scales are worded in easy to understand language and are not focused on any particular disease. The scales are easy to administer and can even measure change in knowledge that occurs during infancy. However, the correlation between CRK and CRP as well as Baby outcome needs further confirmation.

## Conclusion

The CRKS and CRPS were found to be valid reliable instruments to measure maternal child rearing knowledge and practices of women with epilepsy. These scales have potential application in clinical research.

### What is already known

Comprehensive scales to measure child rearing knowledge and practices of mothers with infants are not available.

### What this study adds

We have validated a new set of instruments to evaluate the child rearing knowledge and practice under the four major domains of child rearing: feeding, meeting the needs of cleaning, and protection including prevention of accidents and injuries, providing appropriate infant stimulation, and monitoring growth and development.

## Appendix 1

### Child Rearing Knowledge Scale (CRKS)

**Test to assess the knowledge of mothers regarding child rearing**

Dear participant,

I am glad that you have kindly consented to participate in this study of *child rearing* knowledge and *practice* of mothers. You may be quite excited about pregnancy and caring for your newborn. We would like to provide you some information and skills to improve your child rearing ability. The following questionnaire is designed to evaluate your knowledge on various aspects of child rearing It has twenty questions which may take about 20 min to complete. Each question has five responses. You may select the response that you think is most appropriate for each question. Encircle the alphabet that corresponds to the best response (a,b,c…) of the given responses. If you are unsure about the answers of any question, mark the alphabet for “don’t know.” Do not leave any questions unanswered. Select the nearest accurate response. Please do not seek assistance from anyone to complete it. The right answers will be told to you upon completion of the test. Take your own time. You need not hurry to complete this.

Thank you for your cooperation

**Investigator**

**Table:**

No.	Questions		Best response
1.	The first food to be given to a newborn baby is		c
	a. Boiled and cooled water	b. Glucose water	
	c. Breast milk	d. Any other liquid	
	e. Don’t know		
2.	When should breastfeeding be initiated after a normal delivery?		a
	a. Within half an hour	b. Within 1 hour	
	c. Within 2 hours	d. Within 4 hours	
	e. Don’t know		
3.	How long should breastfeeding be continued?		c
	a. Up to 6 months	b. Up to 1 year	
	c. Up to 2 years	d. As long as the baby needs	
	e. Don’t know		
4.	Which of the following breast parts should be inside the baby’s mouth in correct sucking position?		c
	a. Nipple alone	b. Nipple and part of areola	
	c. Nipple and most of areola	d. Whole breast	
	e. Don’t know		
5.	What is the correct time for beginning complementary feeding?		b
	a. At 4 months	b. At 6 months	
	c. At 1 year	d. Any age	
	e. Don’t know		
6.	A 1-year-old infant can be given		d
	a. Rice and vegetables	b. Fish and minced meat	
	c. Fruits	d. Any home food	
	e. Don’t know		
7.	When does a baby’s weight become approximately double the birth weight?		b
	a. 3 months	b. 5 months	
	c. 9 months	d. 1 year	
	e. Don’t know		
8.	Normally, when does a baby attain head control?		b
	a. 2 months	b. 4 months	
	c. 6 months	d. 1 year	
	e. Don’t know		
9.	Normally when does a baby start sitting without support?		c
	a. 4 months	b. 6 months	
	c. 8 months	d. 10 months	
	e. Don’t know		
10.	Normally when does a baby start saying syllables like ’da’, ’pa’, etc?		c
	a. 4–6 months	b. 6–8 months	
	c. 8–10 months	d. 12 months	
	e. Don’t know		
11.	How can you safeguard a baby against infections?		d
	a. By immunizing the baby	b. By giving daily bath	
	c. By keeping the baby away from infected persons.	d. All the above	
	e. Don’t know		
12.	How can you ensure safety of the baby while bathing him or her?		e
	a. Protect the nose	b. Check the temperature of bath water	
	c. Protect the ears	d. Dry him quickly	
	e. All the above	f. Don’t know	
13.	What is the ideal way to clean the baby’s clothes?		b
	a. Wash with soap and water and dry in shade		
	b. Wash with soap and water and dry in sunlight		
	c. Wash just like any other clothes		
	d. Always dip in disinfectant solution, before washing		
	e. Don’t know		
14.	Which of the following accidents can occur to an infant?		d
	a. Falls	b. Aspiration	
	c. Drowning	d. All the above	
	e. Don’t know		
15.	How can you prevent possible suffocation in infants?		d
	a. Take care that baby’s nostrils are open while breastfeeding		
	b. Keep woolen toys out of reach of babies		
	c. Keep plastic bags out of reach of babies		
	d. All the above		
	e. Don’t know		
16.	Which one of the following immunizations needs not be repeated during infancy?		a
	a. BCG vaccine		
	b. Oral polio vaccine		
	c. HB vaccine		
	d. DPT		
	e. Don’t know		
17.	If the baby gets diarrhea how will you modify his feeds?		d
	a. Continue breastfeeding	b. Give more fluids	
	c. Give easily digestible food	d. All the above	
	e. Don’t know		
18.	A baby explores the world through		d
	a. Playing with toys	b. Interacting with mother	
	c. Interacting with other family members	d. All the above	
	e. Don’t know		
19.	Which one of the following is most helpful for the mental development of the baby?		d
	a. Breast milk	b. Food given by the mother	
	c. Mother’s love and care	d. All the above	
	e. Don’t know		
20.	Language stimulation can be provided to the baby by		c
	a. Providing toys	b. Providing warmth	
	c. Talking and singing	d. Holding and patting	
	e. Don’t know		

## Appendix 2

### Child Rearing Practice Scale (CRPS)

**Child Rearing Practices when the baby is 3 - 4 months old**

**Interview Schedule**

Introduction.

You may be doing many things for your baby, in the process of rearing him up in the best possible way. List down all the activities you do for your baby (This will create interest in the mother and help in establishing rapport with her before going to the items of CRPS).

Child Rearing Practice Scale (CRPS) – at 3 – 4 months Scoring Key: *Rating Scale Item score: 4=always; 3=oft en; 2= occasionally; 1= rarely Check List Item Score: Yes=1, No=0 unless specified otherwise.

**Table:**

Sl no.	Child rearing practices	Response	Scoring
1.	Were you able to give the first milk (Colostrum) to your baby?	Yes No	Yes = 1; No = 0
2.	Were you able to timely initiate breastfeeding within half an hour if normal delivery/ within 4 h if Caesarian?	Yes No	Yes = 1; No = 0
3.	Are you currently breastfeeding your baby?	Yes No	Yes = 1; No = 0
*4.	What type of milk feeds are you giving to your baby?	Exclusive breast feeds = 4, BF+Bottle feed = 3, Bottle feed only = 2, Bottle feed + animal milk = 1	4, 3, 2, 1
5.	Have you started complimentary feeding (-ve item)?	Yes No	No = 1; Yes = 0
*6.	How often are you able to meet the feeding needs of your baby? Always =4; Often =3; Occasionally =2; Rarely=1	Always = 4; Often = 3; Occasionally = 2; Rarely = 1	4, 3, 2, 1
7.	Are you able to meet the needs of your baby during nighttime?	Yes No	Yes = 1; No = 0
8.	Do you check the weight of your baby?	Yes No	Yes = 1; No = 0
9.	Do you check for normal development of your baby (social smile, head holding etc)?	Yes No	Yes = 1; No = 0
10.	Do you keep the baby clean?	Yes No	Yes = 1; No = 0
*11.	How often do you change the diaper as soon as it is soiled?	Always =4; Often =3; Occasionally = 2; Rarely = 1	4, 3, 2, 1
12.	Do you bathe the baby by yourself?	Yes No	Yes = 1; No = 0
13.	Do you groom and dress the baby by yourself?	Yes No	Yes = 1; No = 0
14.	Do you wash the baby’s dresses with soap and water and dry in sunlight?	Yes No	Yes = 1; No = 0
15.	Do you wash your hands before caring the baby?	Yes No	Yes = 1; No = 0
16.	Do you restrict persons with infection from handling your baby?	Yes No	Yes = 1; No = 0
17.	Did you give immunizations to your baby on scheduled dates?	Yes No	Yes = 1; No = 0
18.	Are you in the habit of covering your baby from cold climate?	Yes No	Yes = 1; No = 0
19.	Is your baby sleeping with you in the same bed/same room during night?	Yes No	Yes = 1; No = 0
20.	Do you keep your baby on a mat on the floor?	Yes No	Yes = 1; No = 0
21.	Have your baby met with any accidents (e.g. falling from the cot/cradle/hand, injury from pointed objects)? (-ve item)	Yes No	No = 1; Yes = 0
22.	Are you able to console your baby when he/she cries?	Yes No	Yes = 1; No = 0
23.	Do you talk to your baby/introduce family members to him?	Yes No	Yes = 1; No = 0
24.	Do you play with your baby using colored / sound making toys?	Yes No	Yes = 1; No = 0
25.	Do you sing songs (Lullaby) for your baby to make him sleep?	Yes No	Yes = 1; No = 0
